# Extracellular miR-574-5p Induces Osteoclast Differentiation via TLR 7/8 in Rheumatoid Arthritis

**DOI:** 10.3389/fimmu.2020.585282

**Published:** 2020-10-14

**Authors:** Anett B. Hegewald, Kai Breitwieser, Sarah M. Ottinger, Fariborz Mobarrez, Marina Korotkova, Bence Rethi, Per-Johan Jakobsson, Anca I. Catrina, Heidi Wähämaa, Meike J. Saul

**Affiliations:** ^1^Department of Biology, Technische Universität Darmstadt, Darmstadt, Germany; ^2^Rheumatology Unit, Department of Medicine, Solna, Karolinska Institutet, Karolinska University Hospital, Stockholm, Sweden; ^3^Institute of Pharmaceutical Chemistry, Goethe Universität Frankfurt, Frankfurt, Germany

**Keywords:** TLR7/8, miR-574-5p, extracellular vesicle (EV), osteoclast differentiation, rheumatoid arthritis

## Abstract

Rheumatoid arthritis (RA) is a chronic autoimmune disease characterized by synovial inflammation and joint destruction. Cell-derived small extracellular vesicles (sEV) mediate cell-to-cell communication in the synovial microenvironment by carrying microRNAs (miRs), a class of small non-coding RNAs. Herein, we report that sEV from synovial fluid promote osteoclast differentiation which is attributed to high levels of extracellular miR-574-5p. Moreover, we demonstrate for the first time that enhanced osteoclast maturation is mediated by Toll-like receptor (TLR) 7/8 signaling which is activated by miR-574-5p binding. This is a novel mechanism by which sEV and miRs contribute to RA pathogenesis and indicate that pharmacological inhibition of extracellular miR-574-5p might offer new therapeutic strategies to protect osteoclast-mediated bone destruction in RA.

## Introduction

Rheumatoid arthritis (RA) is one of the most common systemic autoimmune diseases, characterized by synovial inflammation and the destruction of cartilage and bone (1). The pathogenesis of RA is a consequence of a complex interplay between genetic and environmental risk factors, which lead to the loss of immunological tolerance and the activation of innate and adaptive immune responses. Increased osteoclast (OC) differentiation and activation distrupts bone homeostasis in the course of RA (2) by altering the delicate coupling mechanisms between bone formation and resorption (3, 4).

The synovial fluid of affected joints contain high amounts of extracellular vesicles (EV) (5). These are membrane vesicles of endocytic origin that are actively secreted by almost all cell types into biofluids (6) and may play an important role in the pathogenesis of RA (7). A subpopulation of EV, termed small extracellular vesicles (sEV, 50–200 nm in diameter) (8), are involved in cell-to-cell communication in the microenvironment of arthritic joints (9). Among other biologically active substances, sEV contain microRNAs (miRs), a class of small non-coding RNAs (10). MiRs bind in a sequence specific manner to their target messenger RNA (mRNA) and repress gene expression (11, 12). In addition to this, miRs can also activate gene expression by acting as decoys to RNA-binding proteins, thus antagonizing their functions (13, 14). Recently, an alternative function of extracellular miRs has been described, based on their ability to induce innate immune responses. It was shown that sEV delivered miR-29b and miR-21 can bind and activate toll-like receptor 7/8 (TLR7/8) in human lung cancer (15). However, the detailed mechanism of TLR7/8 activation by miRs is not fully understood and the pathological consequences of this pathway have not been analyzed in RA.

In this study we demonstrate that sEV isolated from the synovial fluid of RA patients significantly enhanced the differentiation of OCs *in vitro*. Moreover, we were able to attribute this effect to sEV delivered miR-574-5p, which significantly increased osteoclastogenesis by activating TLR7/8 signaling. Overall, this study indentified extracellular miR-574-5p as a critical mediator in the pathogeneis of RA and indictes it as a promising new target for RNA therapeutics against bone destruction.

## Materials and Methods

### Cell Lines and Cell Culture Conditions

The human embryonal kidney cell line (HEK 293) (DMSZ, ACC 035) and HeLa (DMSZ, ACC57) cells were cultured in Dulbecco's modified Eagle medium (DMEM, Life Technologies, ThermoFisher Scientific, Waltham, USA) with 10% (v/v) heat-inactivated fetal bovine serum (FBS, Life Technologies, ThermoFisher Scientific, Waltham, USA), 100 U/ml penicillin, 100 μg/ml streptomycin and 1 mM sodium pyruvate (PAA the Cell Culture Company, Cölbe, GER). Synovial fibroblasts (SFs) of RA patients were cultured as described previously (16). This study was approved by the Institutional Ethical Committee (Solna, Stockholm, Sweden ethical number 2009/1262-31/3) and is in compliance with all ethical standards and patients' consent according to the Declaration of Helsinki. Cell culture was carried out in a humidified atmosphere of 5% CO_2_ at 37°C. SFs were seeded at a density of 5 × 10^4^ cells/well in 24-well plates and cultured in DMEM-medium supplemented with EV-free FBS (Gibco, Life Technologies, ThermoFisher Scientific, Waltham, USA), 100 U/ml penicillin 100 μg/ml streptomycin and 1 mM sodium pyruvate for 24 h before they were stimulated with 10 ng/ml ml interleukin (IL)-1β (Sigma-Aldrich, Darmstadt, GER) or tumor necrosis factor α (TNF α) (Immuno Tools, Friesoythe, GER) for further 24 h.

### sEV Purification

One milliliter of cell culture supernatant, 500 μl of 1:2 diluted serum (with phosphate buffered saline, PBS, Sigma-Aldrich, Darmstadt, GER) or synovial fluid from RA patients were centrifuged at 2,000 xg at room temperature for 20 min. The synovial fluid was pre-treated with hyaluronidase (1,500 U/ml; Sigma-Aldrich, Darmstadt, GER) for 15 min at 37°C, before sEV were isolated. The supernatant was centrifuged at 21,000 xg in 1.5 ml polypropylene tubes (Beckman Coulter, Brea, USA) at 4°C for 60 min in a L7-65 ultracentrifuge using rotor 70.1.Ti (Beckman Coulter, Brea, USA) to remove large membrane vesicles. The supernatant was transferred in a new polypropylene tube and centrifuged at 100,000 xg at 4°C for 60 min. The supernatant was discarded. The remaining pellet was resuspended in PBS. Quantity of purified sEV was determined on protein level via UV-Vis spectroscopy to ensure treatment with equal amounts of sEV between sEV-treatment experiments. SEV were used directly or stored at 4°C for not longer than 1 week.

### Transmission Electron Microscopy (TEM)

SEV from human serum, HEK 293 cell culture supernatant or synovial fluid were purified and resuspended in PBS. A drop of purified EV was placed on parafilm and a formvar carbon coated nickel grid (Plano, Wetzlar, GER) was placed on top of the drop for 30–60 min. The samples were fixed with 2% paraformaldehyde (Carl Roth, Karlsruhe, GER) for 10 min and washed three times with MQ. SEV were examined using a Zeiss EM109 electron microscope.

### FACS Analysis

Samples containing sEV were thawed in a water bath for ~5 min at 37°C. After a short vortex, 20 μl of sample were incubated for 20 min in the dark with anti-CD63 FITC (abcam, Cambridge, UK). All samples were measured by flow cytometry on a Beckman Gallios instrument (Beckman Coulter, Brea, USA). Thresholds were set to side scatter in order to increase instrument sensitivity and measurements were performed for 60 s. SEV are presented as number of events positive for CD63, minus background noise which was determined using sEV-free buffers (PBS). Conjugate isotype-matched immunoglobulin (FITC) with no reactivity against human antigens was used as negative control.

### Western Blotting

Twenty to thirty microgram of purified sEV were lysed in radioimmunoprecipitation assay buffer (RIPA), consisting of 50 mM Tris-Cl pH 7.4 (Sigma-Aldrich, Darmstadt, GER), 150 mM NaCl (Sigma-Aldrich, Darmstadt, GER), 1% NP40 (Sigma-Aldrich, Darmstadt, GER), 0.25% Na-deoxycholate (Sigma-Aldrich, Darmstadt, GER), 1 mM phenylmethylsulfonyl fluoride (Sigma-Aldrich, Darmstadt, GER), EDTA-free protease inhibitor (Roche, Basel, CHE). SEV concentration was determined using the NanoDrop ND-1000 spectrophotometer (Thermo Fischer Scientific, Waltham, USA) with absorbance at 280 nm. Western blot analysis was performed according to (17). The membranes were incubated with primary antibodies that recognize CD63, CD9, CD81 and Heat shock protein 70 (Hsp70) (all purchased from System Biosciences, Palo Alto, USA). Membranes then were incubated with infrared dye conjugated secondary antibodies (IRDye®, LI-COR® Bioscience, Lincoln, USA) for 45 min at room temperature. Visualization was carried out using Odyssey Infrared Imaging System (LI-COR® Biosciences, Lincoln, USA).

### Overexpression of miR-574-5p in sEV

The XMIRXpress Lenti system (System Biosciences, Palo Alto, USA) was used to overexpress the level of miR-574-5p in sEV (miR-574-5p oe). As negative control (XMIRXP-NT), which inherits a scrambled control, was also purchased by System Biosciences. Twenty-four hours prior to transfection HEK 293 cells were seeded at a density of 7 × 10^5^ cells/well in a 6-well plate. Two microgram of either negative control or miR-574-5p oe plasmid were transiently transfected using Lipofectamine 2000® (Invitrogen, Karlsruhe, GER). Supernatants were harvested for sEV isolation after 16 h. The overexpression efficiency was analyzed by RT-qPCR.

### RNA Extraction

Total RNA from synovial fibroblasts was extracted using miRNeasy Mini Kit (Qiagen, Hilden, GER) according to the manufacturer's instructions. Residual DNA was removed by on-column DNAse digestion using RNase-Free DNase Set (Qiagen, Hilden, GER). Total RNA from purified sEV was isolated using the phenol/guanidinium thiocyanate (GTC)-based extraction method according to (18). 200 nM of a non-human synthetic cel-miR-39-3p (5′-UCACCGGGUGUAAAUCAGCUUG-3′, Sigma Aldrich, Darmstadt, GER) was added as internal standard to compensate for technical and methodical variations.

### Real-time Quantitative PCR (RT-qPCR)

Intracellular and the extracellular miR quantification were performed using RT-qPCR analysis according to (14, 18). qRT-PCR was performed using the following primer: for miR-16-5p (MS0031493), miR-146a-5p (MS00003535), miR-155-5p (MS00031486), miR-574-5p (MS00043617, all from Qiagen, Hilden, GER). For intracellular miR quantification snRNA U6 was used as endogenous control primer (MS00033740, Qiagen, Hilden, GER) and as control for extracellular normalization we used cel-miR-39-3p (MS00019789, Qiagen, Hilden, GER). According to (14), different mRNA transcripts were analyzed. The following primer pairs were used: Interferon α (IFNα-fwd: 5′-GCAAGCCCAGAAGTATCTGC-3′; IFNα-rev: 5′-CTTGACTTGCAGCTGAGCAC-3′), Interleukin 23 (IL-23-fwd: 5′-GTTCCCCATATCCAGTGTGG-3′; IL-23-rev: 5′-AAAAATCAGACCCTGGTGGA-3′), TNFα (TNFα_fwd: 5′- CCCAGGGACCTCTCTCTAATC-3′; TNFα_rev: 5′- ATGGGCTACAGGCTTGTCACT-3′), IL-1β (IL1-β-fwd: 5′-ACAGATGAAGTGCTCCTTCCA-3′; IL1-β-rev: 5′-GTCGGAGATTCGTAGCTGGAT-3′) or IL-8 (IL-8-fwd: 5′-AGCTCTGTGTGAAGGTGCAG-3′; IL-8-rev; 5′-TGGGGTGGAAAGGTTTGGAG-3′). In all experiments β-Actin (Actin-fwd: 5′-CGGGACCTGACTGACTAC-3′; Actin-rev: 5′-CTTCTCCTTAATGTCACGCACG-3′) was used as endogenous control to normalize variations in cDNA quantities in different samples.

### Live Cell Imaging

Purified sEV were labeled with lipophillic tracer 3,3′-Dioctadecyloxacarbocy-anine perchlorate (DiO, Sigma-Aldrich, Darmstadt, GER) for 15 min at 37°C and applied to CD14^+^ monocytes or HeLa cells which were stained with 5 μg/ml Hoechst 33258 (Sigma-Aldrich, Darmstadt, GER) for 30 min. Pictures were taken every 10 min. Confocal images were acquired using an UltraVIEW VoX spinning disk system (PerkinElmer, Waltham, USA) mounted on a Nikon TI microscope (Nikon, Minato, Japan) and equipped with a climate chamber (37°C, 5% CO_2_, 60% humidity). Images were taken at 1 μm Z increments and acquired with a cooled 14-bit EMCCD camera (1,000 × 1,000 pixel frame transfer EMCCD, 30 fps at full frame 1 × 1 binning 35 MHz readout, 8 × 8 μm pixel size) using Volocity 6.3 (PerkinElmer, Waltham, USA).

### RNase and TritonX 100 Treatment of sEV

Isolated miR-574-5p oe sEV were treated either with 0.05 mg/ml RNase A (Qiagen, Hilden, GER) for 20 min at 37°C or with 1% TritonX 100 (Carl Roth, Karlsruhe, GER) for 10 min at RT and 0.05 mg/ml RNase A for 20 min at 37°C in combination. After RNA extraction, the amount of miR-574-5p was analyzed by RT-qPCR.

### Osteoclast Differentiation

Monocytes were isolated from blood donor buffy coats using Ficoll-Paque™ Plus (GE Healthcare, Chicago, USA) separation and CD14^+^ monocytes were selected using CD14 beads (MiltenyiBiotec, Bergisch-Gladbach, GER) according to the manufacturer's instructions. 1 × 10^5^ cells/well were seeded in a 96-well plate in DMEM and differentiated into macrophages using 25 ng/ml macrophage colony-stimulating factor (M-CSF, Peprotech, Rocky Hill, USA) for 3 days. 50% of the culture medium was changed every 3 days and cells were further matured into OCs by medium supplements of 10 ng/mL M-CSF and 5 ng/mL Receptor Activator of NF-κB Ligand (RANKL, R&D Systems, Minneapolis, USA). The number of OCs was assessed using tartrate-resistant acid phosphatase (TRAP) staining (leucocyte acid phosphatase kit 387A, Sigma-Aldrich, Darmstadt, GER). Number of OCs was counted using a light microscope. TRAP^+^ cells with at least or more than three nuclei were considered as being OCs. Each biological replicate has four technical replicates.

### Stimulation of CD14^+^ Monocytes

CD14^+^ monocytes were isolated and seeded at a density of 1 × 10^5^ cells/well in a 96-well plate and stimulated as indicated with purified EV or Resiquimod - R848 (Invivogen, San Diego, USA), diluted in Tris-EDTA (TE) buffer. Cells were additionally treated with 200 nM of the TLR7/8 inhibitor ODN 2088 Control (2087) (MiltenyiBiotec, Bergisch-Gladbach, GER), diluted in TE buffer, 30 min prior to stimulation with EV or R848 at indicated experiments. Furthermore, cells were treated either with 5 or 50 nM of synthetic miR-574-5p (Sigma-Aldrich, Darmstadt, Germany) together with or without 1 μg/ml Lipofectamine® 2000 (Thermo Fisher Scientific, Waltham, USA).

### Cytokine mRNA Analysis

CD14^+^ monocytes were isolated and seeded at a density of 1 × 10^6^ cells/well in a 12-well plate and stimulated either with 1 μg/ml purified scrambled control or miR-574-5p oe sEV or 10 ng/ml R848 for 4 h. For experiments with the TLR7/8 inhibitor ODN 2088 Control (2087), cells were treated with 200 nM inhibitor 30 min prior to stimulation with sEV or R848 positive control. The mRNA level of each cytokine was analyzed by RT-qPCR.

### Microscale Thermophoresis (MST)

The Cyanine5-labeled hsa-miR-574-5p (5′-UGAGUGUGUGUGUGUGAGUGUGU [Cyanine5]-3′, Sigma-Aldrich, Darmstadt, GER) was adjusted to 100 nM with PBS (pH7.4, Thermo Fisher Scientific, Waltham, USA) supplemented with 0.05% Tween 20 (NanoTemper Technologies, München, GER). A series of 16 1:2 dilutions was prepared with recombinant human TLR8 (H00051311_G01, Abnova, Taiwan) using the protein storage buffer (25 mM Tris-HCl pH 8.2% Glycerin), producing ligand concentrations ranging from 20.4 pM to 668 nM. For the measurement, each ligand dilution was mixed with one volume of labeled hsa-miR-574-5p which leads to a final concentration of hsa-miR-574-5p of 50 nM and final ligand concentrations ranging from 10.4 pM to 334 nM. After 5 min incubation, the samples were loaded into Monolith NT.115 Standard Treated Capillaries (NanoTemper Technologies, München, GER). Instrument parameters were adjusted to 60% LED power and 40% MST power. A negative control was performed using Cy5-labeled hsa-miR-16 (5′-UAGCAGCACGUAAAUAUUGGCG[Cyanine5]-3′, Sigma-Aldrich, Darmstadt, GER) with a final concentration of 50 nM and a final TLR8 concentration ranging from 6.36 pM to 209 nM. Instrument parameters were adjusted to 20% LED power and 60% MST power. Data of three independently measurements were analyzed (MO.Affinity Analysis software version 2.1.3, NanoTemper Technologies, München, GER) using the signal from an MST-on time of 1.5 s.

### Immunofluorescence

1 × 10^6^ CD14^+^ cells were seeded on poly-L-lysine (Sigma-Aldrich, Darmstadt, GER) coated glass coverslips (12 mm, Neolab, Heidelberg, GER) allowed to settle for 24 h. Cells were fixed with 4% formaldehyde (FA, Carl Roth, Karlsruhe, GER) for 10 min. After washing 3 times for 3 min with PBS, cells were permeabilized with 0.5% Triton X-100 (Sigma-Aldrich, Darmstadt, GER) in PBS for 10 min. Subsequently, cells were blocked with 4% BSA (Sigma-Aldrich, Darmstadt, GER) in PBS for 20 min. The primary antibodies against TLR7 (MA5-16247, Invitrogen, ThermoFisher Scientific, Waltham, USA) or TLR8 (PA5-20056, ThermoFisher Scientific, Waltham, USA) were diluted 1:50 (TLR7) or 1:100 (TLR8) in blocking solution and incubated for 1 h at room temperature. Afterwards, cells were washed with 0.01% Tween20 (Carl Roth, Karlsruhe, GER) in PBS 3 times for 5 min and incubated either with secondary antibody goat anti-mouse IgG (Alexa Fluor® 594, ab150116, abcam, Cambridge, UK) diluted 1:500 for TLR7 or goat anti rabbit IgG (Alexa Fluor® 488, 111-454-144, JacksonImmunoResearch, Ely, UK) in blocking solution for 45 min at room temperature. Finally, cells were washed with 0.01% Tween20 (Carl Roth, Karlsruhe, GER) in PBS and counterstained with 4′,6-diamidine-2′-phenylindole dihydrochloride (DAPI, Sigma-Aldrich, Darmstadt, GER) for 5 min and mounted in 1,4-Diazabicyclo[2.2.2]octane (DABCO, Sigma-Aldrich, Darmstadt, GER). Fluorescence microscopy was carried out using a Zeiss Axiovert 200 microscope. Images were taken with Plan-Apochromat 100x/NA 1.4 (pixel size XY = 66 nm) oil immersion objective and a Zeiss AxioCam mRM camera.

### Fuorescences *in-situ* Hybridization of miR-574-5p

2.5 × 10^5^ CD14^+^ monocytes were seeded on 12 mm glass coverslips in a 24-well plate and differentiated into M2-like macrophages by addition M-CSF to a final concentration of 25 ng/ml and further cultivation for 3 days.

Cells were fixed in formaldehyde (4% in PBS, 10 min at room temperature) followed by Trition X-100 permeabilization (0.5% in PBS, 10 min at room temperature), samples were washed three times with PBS containing 0.01 % Tween20, respectively. Coverslips where then placed at 37°C for 30 min covered with 1x miR ISH buffer (Qiagen, Hilden, GER, Cat No./ID: 339450) for prehybridization. Coverslips where then transferred into a hybridization chamber and covered with double DIG labeled anti miR-574-5p probe (Qiagen, Hilden, GER, 100 nM in 1x miR ISH buffer). Hybridization was carried out at 54°C for 1 h. Samples were transferred into a humidified staining chamber and washed twice with 54°C warm 2x SSC buffer (20x SSC stock solution, Invitrogen Karlsruhe, GER). Blocking was performed with 2% BSA (in PBS, 20 min at room temperature). Antigen detection was performed over night at 4°C. Anti-miR-574-5p DIG probe was detected by rabbit anti-DIG antibodies (Thermo Fisher Scientific, Waltham, USA, 9H27L19, 1 ng/ml final, diluted in blocking buffer) and TLR8 was detected by mouse anti-TLR8 antibodies (Thermo Fisher Scientific, Waltham, USA, 44C143, 1 ng/ml final, diluted in blocking buffer). Samples were washed three times with PBS containing 0.01% Tween20. Primary antibodies where detected using donkey anti-rabbit AlexaFluor 594 conjugated antibodies (abcam, Cambridge, UK, ab150080, 5 ng/ml final, diluted in blocking buffer) and donkey anti-mouse AlexaFluor 488 conjugated antibodies (abcam, Cambridge, UK, ab150105, 5 ng/ml final, diluted in blocking buffer) for 1 h at room temperature. Samples were washed again three times with PBS containing 0.01% Tween20. Nuclei where counterstained using DAPI (Sigma-Aldrich, Darmstadt, GER, 1 μg/ml, 10 min at room temperature). Samples were washed once with water and mounted using Mowiol (Sigma Aldrich, Darmstadt, GER).

### Statistics

Results are presented as mean + SEM or ± SD. Statistical analysis was carried out using Student's unpaired *t*-test (two-tailed) or one-way ANOVA with turkey post-test using GraphPad Prism 6.0. Differences were considered as significant for *p* < 0.05 (indicated as ^*^ for *p* < 0.05, ^**^ for *p* < 0.01, ^***^ for *p* < 0.001 and ^****^ for *p* < 0.0001). Dixon's test was performed as outlier test.

## Results

### Isolation of sEV From Synovial Fluid of RA Patients

**Figure 1:**
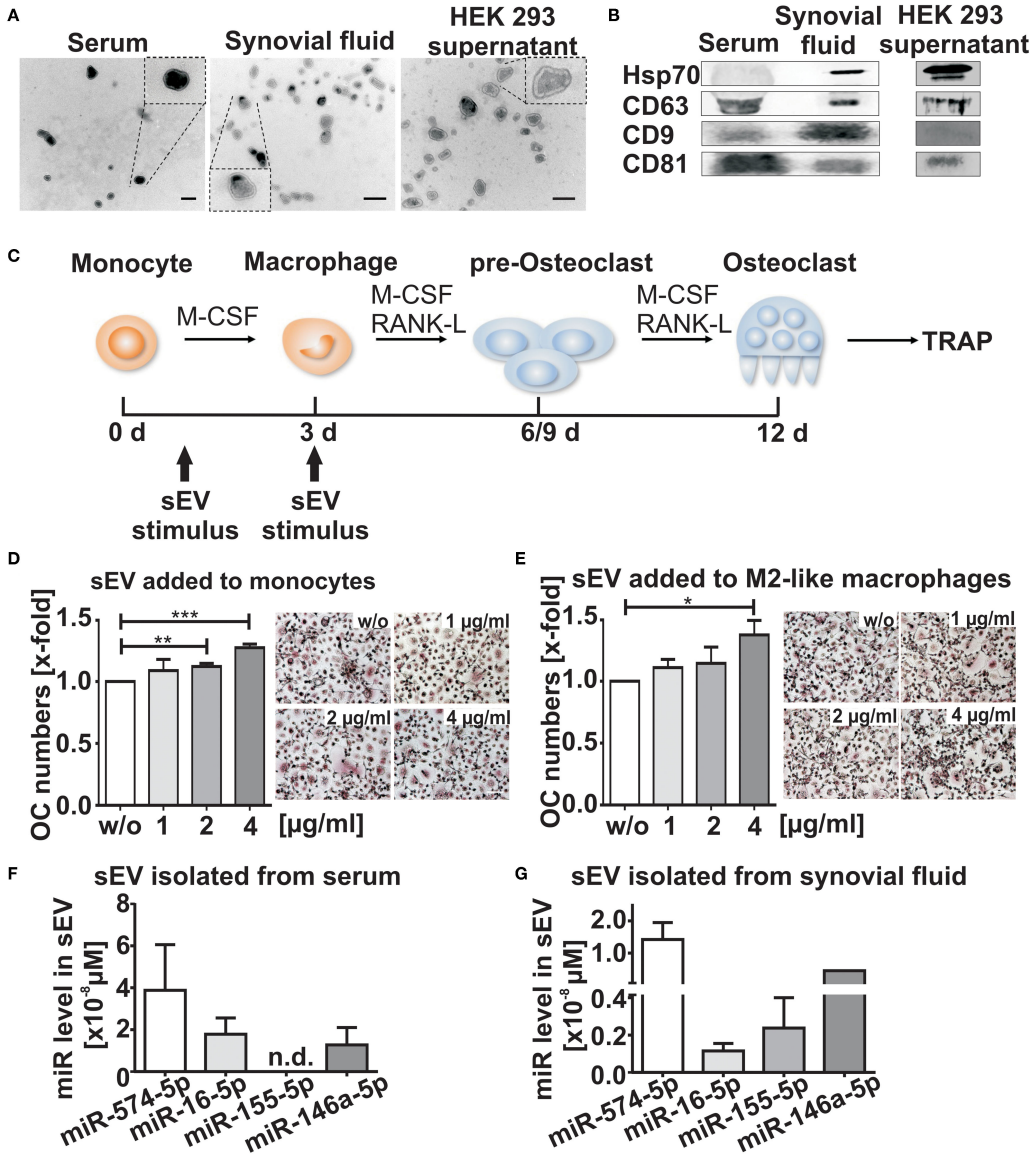
Characterization of small extracellular vesicles (sEV). sEV were isolated by differential ultracentrifugation from serum, synovial fluid and HEK 293 cell supernatants. **(A)** Transmission electron microscopy (TEM), scale bar 200 nm. **(B)** Western blot analysis of CD9, Hsp70, CD63, and CD81 (*N* = 3). Blots and TEM pictures are shown from one representative experiment. **(C)** Schematic diagram of osteoclast (OC) differentiation. Cells were stimulated with sEV either at the monocyte stage (day 1) or the M2-like macrophage stage (day 3) as indicated by the arrows **(D,E)** Tartrate-resistant acid phosphatase (TRAP) staining of mature OCs obtained from CD14^+^ monocytes and cultured in presence of sEV derived from synovial fluid of two ACPA^+^ RA patients (1, 2, or 4μg/ml). sEV were added to **(D)** CD14^+^ monocytes and to **(E)** M2-like macrophages. Multinucleated cells with three or more nuclei that were stained with a purple color were considered as OCs. The relative changes are given as mean + SEM (*N* = 4), *t*-test **p < 0.01, ***p < 0.001, scale bar 50 μm. Representative images of TRAP positive cells are shown. **(F,G)** sEV miR level of miR-574-5p,−16-5p,−155-5p, and−146a-5p isolated from 500 μl serum **(F)** or 500 μl synovial fluid **(G)** from three RA patients. Before sEV isolation, synovial fluid was pre-treated with hyaluronidase (1,500 U/ml) for 15 min at 37°C. MiR levels were determined by RT-qPCR and normalized to the spike-in control cel-miR-39-3p (200 nM). Data are shown as the mean + SEM (*N* = 3). *p < 0.05.

We isolated sEV from the synovial fluid of RA patients positive for anti-citrullinated protein antibodies (ACPA^+^), which is associated with a more aggressive RA disease course and enhanced bone resorption (19). The vesicles were isolated using differential ultracentrifugation (Supplementary Figure 1A). The morphology and the size of the isolated vesicles were determined by TEM, showing that the isolated population had the typical vesicular morphology and sEV size, ranging from 50 to 150 nm (Figure 1A). Using Western blotting, we were able to detect specific sEV surface protein markers such as CD63, CD9, Hsp70, and CD81 in sEV lysates (Figure 1B) (8).

### sEV Derived From Synovial Fluid of RA Patients Induce Osteoclastogenesis

We next investigated whether sEV isolated from the synovial fluid of ACPA^+^ RA patients influence osteoclastogenesis. CD14^+^ monocytes were separated from peripheral blood mononuclear cells of healthy donors and stimulated with recombinant human M-CSF, RANKL (20) in combination with different concentrations of sEV (Figure 1C). In order to assess whether there were time- and maturation stage-related differences, both freshly isolated CD14^+^ monocytes and M2-like macrophages (21) were treated with sEV (Figures 1D,E). After 9–12 days, cells were fixed and stained for the OC marker TRAP. We observed a significant dose-dependent increase (about 30%) in OC numbers, when the sEV were applied to monocytes (Figure 1D). A similar increase was observed, when macrophages, a later stage of OC differentiation, were treated with sEV (Figure 1E). These results suggest that the content of sEV derived from the synovial fluid of RA patients may contribute to an increased OC differentiation process.

### Synovial Fluid Derived sEV Contain High Levels of miR-574-5p

It is known that miRs are selectively packaged in extracellular vesicles (22) and modulate inflammation and OC formation (23). Therefore, we selected four different miRs (hsa-miR-146a-5p, miR-155-5p, miR-16-5p and miR-574-5p) which previously have been associated with inflammation and/or osteoclastogenesis (14, 24–28). RT-qPCR was performed to analyze the level of each miR in sEV isolated from synovial fluid and in the corresponding serum samples from three RA patients. In contrast to miR-146a-5p, miR-16-5p and miR-155-5p, high levels of miR-574-5p were detected in sEV isolated from synovial fluid and serum samples. Notably, miR-146a was only detected once in sEV isolated from synovial fluid, while the same miR was consistently present in serum-derived sEV (Figures 1F,G).

### Synovial Fibroblasts as Cellular Source of sEV Derived miR-574-5p

**Figure 2:**
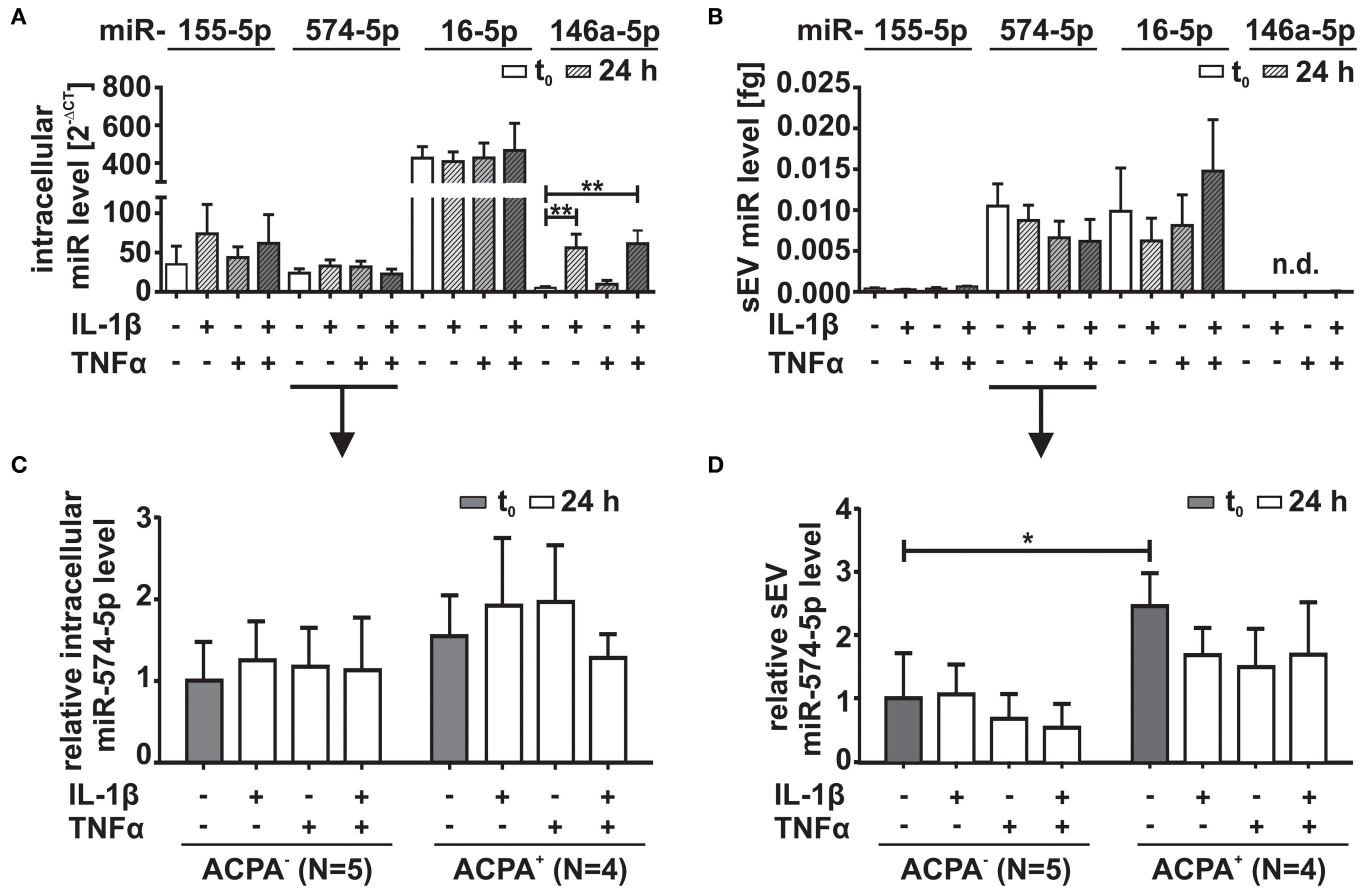
Comparison of miR-574-5p, 16-5p, 155-5p, and 146a-5p levels in RA synovial fibroblasts (SFs). SFs from RA patients were cultured in sEV-depleted cell culture medium for 24 h. Medium was replaced by sEV-depleted cell culture medium supplemented with either 10 ng/ml IL-1β, 10 ng/ml TNFα or both for 24 h. MiR levels were determined by RT-qPCR. **(A)** Intracellular miR levels were normalized to snRNA U6 endogenous control. Data are shown as mean + SEM (*N* = 10), *t*-test **p < 0.01. **(B)** MiR content in sEV derived from 1 ml cell supernatant was determined by RT-qPCR normalized to the spike-in control cel-miR-39-3p (200 nM). Data is shown as the mean + SEM of (*N* = 9). The results of **(C)** intracellular and **(D)** extracellular miR-574-5p were additionally evaluated with respect to patient characteristics. Therefore, miR-574-5p levels of SFs cells derived from ACPA^−^ RA patients (*N* = 5) or RA patients (*N* = 4) were normalized to the mean of ACPA^−^ patients. The relative changes are shown as the mean + SEM. *p < 0.05.

SFs play a crucial role in the pathogenesis of RA (29, 30). We therefore analyzed intracellular and corresponding sEV levels of miR-574-5p released from RA derived SFs using RT-qPCR and compared those to the levels of miR-146a-5p,−155-5p and−16-5p. In order to mimic the inflammatory environment of the inflamed joint, we stimulated SFs with 10 ng/ml IL-1β or 10 ng/ml TNFα alone or in combination for 24 h (Figures 2A,B). We observed that miR-155-5p, miR-16-5p, or miR-574-5p levels did not change significantly in response to IL-1β or TNFα stimulation. MiR-146a-5p was included in our experiments as a positive control. This miR has been reported to be strongly induced by IL-1β stimulation which our experiments also confirmed (31). TNFα stimulation alone had no influence on miR-146a-5p expression (Figure 2A). We next isolated sEV from SF cell culture supernatants and performed RT-qPCR. Very low levels of miR-155-5p or no miR-146a-5p were found in sEV regardless of stimulation. In contrast, high amounts of miR-16-5p and miR-574-5p were detected in the sEV purified from both cytokine stimulated and unstimulated SFs (Figure 2B), indicating that SFs are competent to secrete miR-574-5p containing sEV in RA joints. In concordance with a recent publication (32), we observed a slight but significant increase in the number of sEV in response to both IL-1β and TNFα stimulation (Supplementary Figures 1B,C).

Our next step was to compare intra- and extracellular miR-574-5p levels with regard to patients' ACPA status. No significant differences were evident comparing the intracellular expression of miR-574-5p in unstimulated SFs (Figure 2C). Contrary, higher concentrations of miR-574-5p were observed in sEV generated by SFs from ACPA^+^ compared to ACPA^−^ patients (Figure 2D).

However, the extracellular level of miR-574-5p was significantly higher in sEV derived from unstimulated SFs from ACPA^+^ RA patients compared to SFs isolated from ACPA^−^ patients (Figure 2D).

### Overexpression of miR-574-5p in sEV

**Figure 3:**
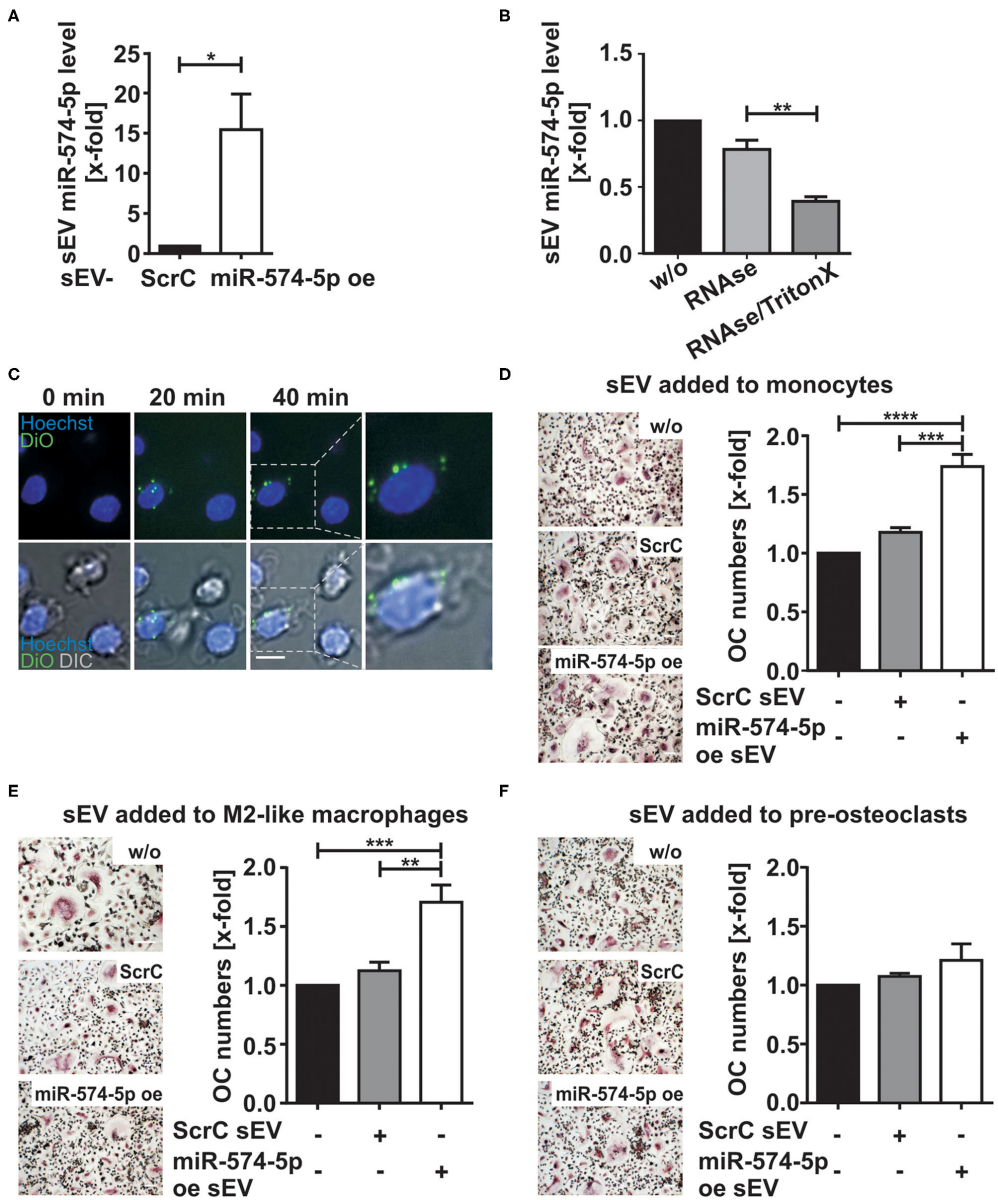
Effect of sEV delivered miR-574-5p on OC differentiation. **(A)** HEK 293 cells were transfected with scrambled control (ScrC) or over expressed miR-574-5p (miR-574-5p oe) XMIRXpress construct. The sEV miR-574-5p level was analyzed by RT-qPCR and normalized to the spike-in control cel-miR-39-3p (200 nM). Relative changes to ScrC control are presented as the mean + SEM (*N* = 4), *t*-test *p < 0.05. **(B)** miR-574-5p oe sEV were treated with 0.05 mg/ml RNase A with and without 1% TritonX. MiR-574-5p level was determined by RT-qPCR and normalized to the spike-in control cel-miR-39-3p. Relative changes to untreated sEV (w/o) are shown as the mean + SEM (*N* = 3), *t*-test **p < 0.01. **(C)** Purified miR-574-5p oe sEV were labeled with lipophilic tracer 3,3′-dioctadecyloxacarbocy-anine perchlorate (DiO) and added to CD14^+^ monocytes, which were stained with 5 μg/ml Hoechst 33258. Images were taken every 10 min, scale bar 10 μm. **(D–F)** TRAP staining of mature OCs obtained from CD14^+^ monocytes. The cells were cultured in presence of 1 μg/ml ScrC or miR-574-5p oe sEV, which were added at **(D)** monocyte stage, **(E)** M2-like macrophage stage or **(F)** pre-osteoclast stage. TRAP-positive cells with three or more nuclei were considered as OCs. The relative changes normalized to untreated control cells are given as mean + SEM **(D,E)** (*N* = 6) and **(F)** (*N* = 3), one-way ANOVA **p < 0.01, ***p < 0.001, ****p < 0.001, scale bar 50 μm.

To assess whether the elevated miR-574-5p level in sEV had an influence on osteoclastogenesis, we established a miR overexpression system in HEK 293 cells that enhanced miR-574-5p loading into sEV (miR-574-5p oe sEV). For control experiments, we used sEV loaded with a scrambled miR (ScrC sEV). Comparing oe sEV with ScrC sEV (Figure 3A), we detected a ~15-fold increase of miR-574-5p in the oe sEV. A RNase protection experiment was performed by treating the sEV with RNase alone or together with a detergent to determine if miR-574-5p was loaded into the sEV or if it was only attached to the sEV surface (33). The miR-574-5p was protected from RNase I digestion unless detergent was added to disrupt the membrane (Figure 3B). Therefore, we conclude that at least the majority of miR-574-5p is selectively loaded into the oe sEV. Finally, we could show that these sEV are taken up by cells like monocytes (Figure 3C) and HeLa cells (Supplementary Figure 1D), using confocal microscopic live cell imaging.

### sEV With High miR-574-5p Levels Induce Osteoclastogenesis

In order to investigate the role of sEV derived miR-574-5p during osteoclastogenesis, we added either 1 μg/ml miR-574-5p oe sEV or ScrC sEV at different time points during OC differentiation (Figures 3D–F). After ~12 days of differentiation, the cells were TRAP-stained and the number of OCs was counted.

When sEV were added at the stage of monocytes, a significant upregulation of OC numbers was observed in response to miR-574-5p oe sEV compared to ScrC sEV or untreated control (Figure 3D). We observed comparable results, when the engineered sEV were added at the stage of macrophages (Figure 3E). No significant changes in OC numbers were found when sEV were added to pre-OCs, neither with the addition of control or miR-574-5p oe sEV (Figure 3F). These results suggest that the effect of extracellular miR-574-5p strongly depends on the maturation stage of the cells during OC differentiation.

### Increased Osteoclast Differentiation by miR-574-5p Is Mediated by TLR7/8

**Figure 4:**
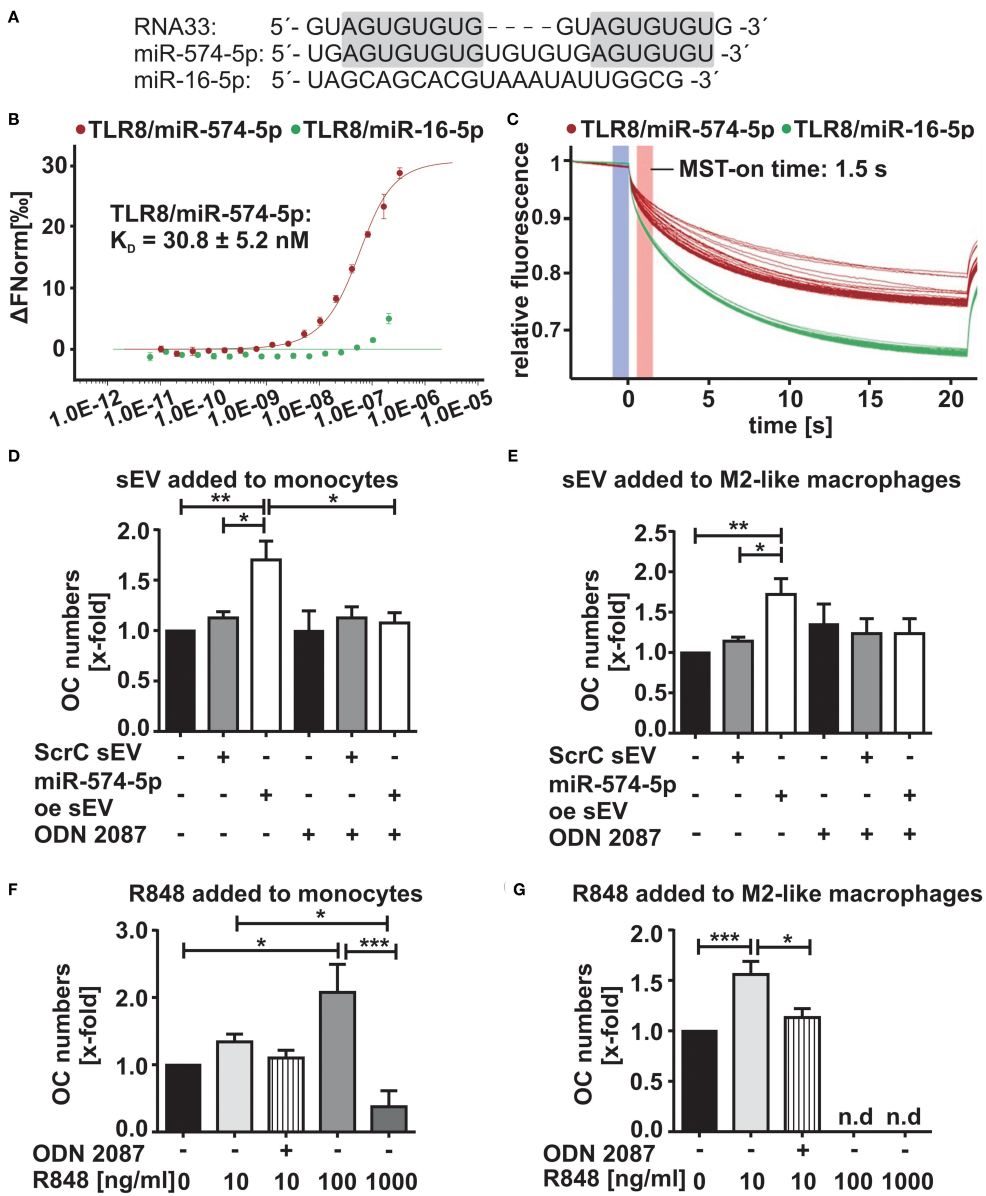
Increased OC differentiation by sEV-delivered miR-574-5p is mediated by TLR7/8 activation **(A)** Comparison of sequence homologies of miR-574-5p and miR-16-5p to RNA33. **(B,C)** In microscale thermophoresis (MST) affinity assay of miR-574-5p to human recombinant TLR8, 50 nM of Cy5-labeled miR-574-5p and 334 nM−10.2 pM of non-labeled TLR8 were used. As negative control, 50 nM of Cy5-labeled hsa-miR-16-5p with 209 nM−6.36 pM of TLR8 were used. After a short incubation, samples were loaded into Monolith NT.115 Standard Treated Capillaries and the MST measurement was performed **(B)** Dose response curve reveals a K_D_ of 30.8 ± 5.24 nM for the interaction of miR-574-5p and TLR8. Data are shown as ± SD (*N* = 3). **(C)** MST traces shown for miR-574-5p or miR-16-5p and TLR8. An MST-on time of 1.5 s was used for analysis. **(D–G)** TRAP-positive OCs obtained from CD14^+^ monocytes cultured in presence of **(D,E)** 1 μg/ml ScrC- or miR-574-5p oe sEV together with 200 nM TLR7/8 inhibitor ODN 2087 or **(F,G)** 10–1,000 ng/ml - TLR7/8 ligand R848 added either at (D/F) monocyte stage or (E/G) M2-like macrophage stage. TRAP-positive cells with three or more nuclei were considered as OCs. The relative changes normalized to untreated control cells are given as mean + SEM (*N* = 4), one-way ANOVA, *p < 0.05, **p < 0.01, ***p < 0.001.

First hints indicating that miR-574-5p represents a novel TLR7/8 agonist (34) were based on its sequence homology to RNA33, a well-known TLR7/8 ligand (35) (Figure 4A). To validate a direct interaction between miR-574-5p and TLR8, MST (36) was performed using Cy5-labeled miR-574-5p and commercially available human recombinant TLR8. We observed strong binding of miR-574-5p to TLR8 with a dissociation constant (K_D_) of 30.8 ± 5.24 nM (Figures 4B,C). As negative control we used miR-16-5p, a randomly chosen natural miR that contains no binding sequence for TLR8 (Figure 4A), which shows no binding capacity in MST analysis (Figures 4B,C).

We next investigated whether the increased OC differentiation was due to the activation of TLR7/8. During OC differentiation, all cell types were stained positively for TLR7/8 expression (Supplementary Figures 2A,B). Furthermore, occasional colocalization between miR-574-5p and TLR8 was observed in M2-like macrophages (Supplementary Figures 2C,D). Therefore, we isolated and stimulated monocytes and macrophages with miR-574-5p oe sEV or ScrC sEV together with 200 nM of the TLR7/8 inhibitor ODN 2087. As expected, the miR-574-5p-mediated effect was completely blocked by the addition of the inhibitor (Figures 4D,E; Supplementary Figures 3A,B). As a control experiment we used R848, a known TLR7/8 ligand (37, 38), which was added to the monocytes and macrophages instead of sEV at a concentration of 10 ng/ml in the same experimental setup. As previously with miR-574-5p oe sEV, a significant increase of OC numbers was observed (Figures 4F,G; Supplementary Figures 3C,D) that was blocked using TLR7/8 inhibitor. No increase in osteoclastogenesis was observed, when pre-OCs were stimulated with the TLR7/8 agonist (Supplementary Figure 3E). This result was consistent with the results with miR-574-5p oe sEV or ScrC sEV (Figure 3F). Notably, we observed an increase in OC differentiation, when monocytes or macrophages were stimulated with the TLR7/8 ligand R4848. While the agonist led to an increase in OC numbers at low doses, the opposite effect was observed at higher R848 concentrations. The number of OCs was drastically reduced, when cells were treated with 1 μg/ml R848 at all differentiation time points. The same negative effect was observed when 100 ng/ml of R848 were added to macrophages and pre-OCs (Figures 4F,G; Supplementary Figure 3E). In summary, our results strongly suggest that the increase in OC differentiation by sEV derived miR-574-5p is mediated by TLR7/8 activation.

### sEV Delivered miR-574-5p Induces IFNα and IL-23 mRNA in CD14+-monocytes via TLR7/8 Activation

**Figure 5:**
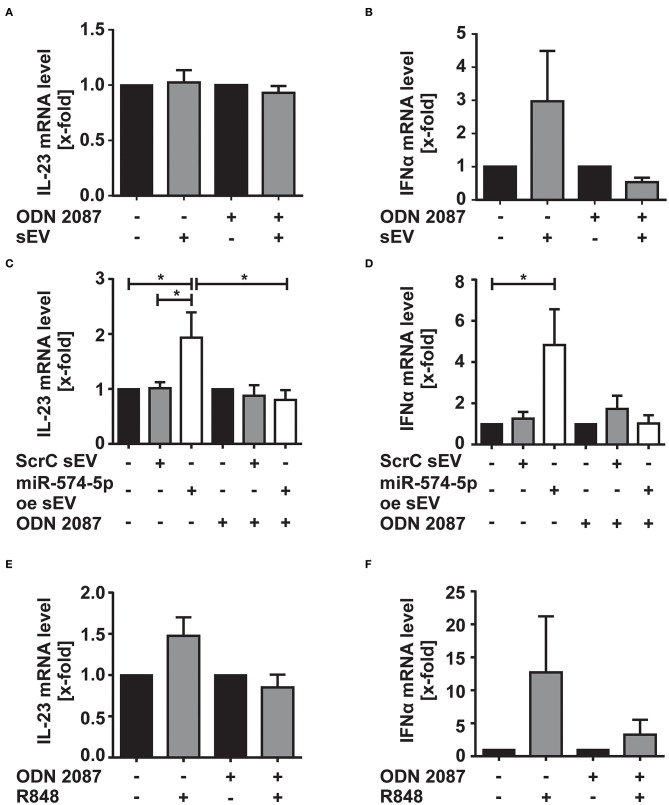
Effect of sEV-delivered miR-574-5p on IL-23 and IFNα mRNA levels. CD-14+ monocytes were stimulated with **(A,B)** 4 μg/ml sEV isolated from synovial fluid of ACPA+ RA patients or **(C,D)** 1 μg/ml of ScrC or miR-574-5p oe sEV and 200 nM ODN 2087. Cells were harvested after 4 h of incubation and total RNA was extracted. Quantification of **(A,C)** IL-23 and **(B,D)** IFNα mRNA levels using RT-qPCR. **(E,F)** CD14+ monocytes were stimulated with 10 ng/μl R848 and 200 nM of ODN 2087 for 4 h. Total RNA was extracted, and RT-qPCR was performed to quantify **(E)** IL-23 and **(F)** IFNα mRNA level. β-Actin was used as endogenous control. Relative changes normalized to untreated controls are given as + SEM (*N* = 4), one-way ANOVA, *p < 0.05.

We next aimed to understand which cytokines were increased by sEV with high miR-574-5p levels. Therefore, we stimulated CD14^+^ monocytes either with 4 μg/ml of sEV isolated from the synovial fluid of ACPA^+^ RA patients or with 1 μg/ml of miR-574-5p oe sEV or ScrC sEV for 4 h. Total RNA was isolated and the mRNA levels of different TLR7/8 target genes such as IL-23, IL-8, INFα, IL-1β, and TNFα were analyzed (Figure 5; Supplementary Figure 4). These cytokines are known to influence OC differentiation (39). We observed no changes in IL-23 mRNA levels, while IFNα mRNA levels increased about ~3-fold, when cells were treated with sEV isolated from synovial fluid (Figures 5A,B). When stimulating the monocytes with miR-574-5p oe sEV, we observed significant 2- and 5-fold inductions of IL-23 and IFNα mRNA, respectively, while ScrC sEV had no effect (Figures 5C,D). The induction of IL-23 and IFNα was reversed by additional application of the TLR7/8 inhibitor. Comparable results were obtained when monocytes were stimulated with 10 ng/ml of the TLR7/8 ligand R848 (Figures 5E,F). In contrast to IL-23 and IFNα, the mRNA levels of TNFα, IL-1β, and IL-8 were not significantly affected by sEV isolated from the synovial fluid of RA patients (Supplementary Figures 4A–C), miR-574-5p oe sEV or R848 (Supplementary Figures 4D–I).

## Discussion

sEV were identified as a key factor in cell-to-cell communication through transfer of miRs (10, 22). A better understanding of their physiological function in the synovial microenvironment of RA patients is essential for the development of novel treatment strategies. While previous studies have shown that extracellular vesicles play an important role in the pathogenesis of RA (40), the molecular mechanism and their impact on bone resorption needs to be further elucidated. Our study shows for the first time that sEV isolated from synovial fluid of RA patients caused an increased OC differentiation, which we attribute to high abundance of miR-574-5p in the sEV.

Different cell types in the joint microenvironment can contribute to aggressive cartilage and bone resorption (1). In particular, activated SFs strongly induce osteoclast formation (29, 30). We identified SFs as a cellular source of sEV with high miR-574-5p content. Despite the low number of patient-derived synovial fibroblasts, we observed higher levels of miR-574-5p in the sEV derived from ACPA^+^ compared to ACPA^−^ RA patients. Since ACPA positivity is associated with a more severe and aggressive course of RA (19, 41), it is intriguing to propose that miR-574-5p might play a role in ACPA^+^ RA. However, it is for future studies to identify the source of high miR-574-5p levels in ACPA^+^ RA patients and to investigate the potential impact of ACPAs on miR-574-5p secretion into sEV.

In order to perform functional assays, we established an overexpression system in which sEV were loaded with high amounts of miR-574-5p. We demonstrated that miR-574-5p oe sEV were taken up by cells and were physiologically active. CD14^+^ monocytes and M2-like macrophages were stimulated with miR-574-5p oe sEV and significant changes in OC formation were observed compared to controls. No changes were observed when the same sEV were applied to pre-OCs, indicating that only a certain progenitor cell stage is responsive to extracellular miR-574-5p. Although all cell types show TLR7/8 expression, it can be speculated that certain downstream proteins are induced in pre-OCs which negatively regulate TLR signaling in response to miR-574-5p stimulation (42).

Furthermore, it suggests that the sEV delivered miR-574-5p might have immune-modulating functions. miRs have proven to be regulators in immune response (43) via binding to TLRs (15, 44, 45). sEV-delivered miR-let-7b is able to transform human- and mouse naive monocytes into inflammatory M1-like macrophages by activating TLR7 (44). This highlights the importance of extracellular miRs in cell-to-cell communication and their impact on chronic inflammatory diseases. However, the mechanisms of extracellular miR release and their effects on target cells are not fully understood.

In our experimental setup, we demonstrated that miR-574-5p induces osteoclastogenesis only when loaded into sEV. Synthetic liposomal vehicles or synthetic miRs alone had no effect on osteoclastogenesis (Supplementary Figures 5A,B). These findings are in agreement with current understanding that vesicle-associated miRs are important players in the cell-to-cell communication, while free miRs may only represent cell by-products without physiological impact. We can speculate that sEV uptake depends on proteins and glycoproteins expressed on the surface of the vesicle as well as on the surface of the target cell (46). Furthermore, Fabbri et al., demonstrated that extracellular vesicle-delivered miR-21 and−29a bind to human TLR8 and trigger downstream to NF-κB activation in the context of non-small cell lung cancer (15). Since miR-574-5p has a high sequence analogy to the TLR7/8 ligand RNA33 and induced phosphorylation of the p65 subunits of NF-κB (15, 34, 35), we asked whether miR-574-5p might act as direct TLR7/8 ligand and increases OC differentiation by TLR7/8 activation. With the results of our MST binding test and our experiments using the TLR7/8 inhibitor ODN 2087 and the agonist R848, we could show that miR-574-5p binds to the receptor and thus mediates increased OC formation via TLR7/8. However, our results seem to contradict a previous study by Miyamoto et al., which reports a decrease in OC formation in response to R848 stimulation (47). This inconsistency could be explained by slight experimental differences such as the R848 concentration and the cell types used to analyze the OC maturation process. Our results indicate that sEV delivered miR-574-5p has the highest influence on osteoclastogenesis at monocyte and M2-like macrophage stages compared to the pre-OC stage. This is probably due to the binding of miR-574-5p to TLR7/8 whose expression level is reduced during the monocyte differentiation process (48). In addition, our results are supported by findings of Salvi et al., who showed that extracellular miR-574-5p can promote production of IFNα by inducing human TLR7 activation in human plasmacytoid dendritic cells (34). In fact, we observed similar results when we treated monocytes with miR-574-5p oe sEV stimulation or TLR7/8 ligand R848. In both cases, we observed an increase in IFNα and IL-23 mRNA levels. Extracellular miR-574-5p can therefore be considered as a new immune-modulating mediator which strongly influences bone resorption in RA via its function as TLR7/8 ligand.

Additionally, it has recently been shown that an elevated intracellular miR-574-5p expression is directly associated with an enhanced synthesis of prostaglandin E_2_ (14), an important pro-inflammatory lipid mediator which mediates inflammation in RA (49). This newly discovered link between miR-574-5p, inflammation and OC-mediated bone resorption offers the opportunity to develop new RNA-therapeutics. Inhibitors against miR-574-5p would address simultaneously its intracellular function as a regulator of prostaglandin synthesis and its endosomal function as TLR7/8 ligand, which would inhibit bone resorption in arthritis disease such as RA.

## Data Availability Statement

All datasets presented in this study are included in the article/supplementary material.

## Ethics Statement

The studies involving human participants were reviewed and approved by the Institutional Ethical Committee (Solna, Stockholm, Sweden, the ethical permit number 2009/1262-31/3) and is in compliance with all ethical standards and patients' consent according to the Declaration of Helsinki. The patients/participants provided their written informed consent to participate in this study.

## Author Contributions

AH performed the biochemistry experiments, analyzed the data, and wrote the manuscript. KB performed the colocalization staining, supported the experiments, and contributed to writing. SO performed the MST analysis and supported the experiments. FM performed the FACS analysis. MK, P-JJ, AC, and HW provided patient material. BR and HW designed and supported the experiments. HW contributed to writing. MS conceived the study, designed and supervised the overall project, and wrote the manuscript. All authors conducted the quality assurance of the paper and reviewed the manuscript.

## Conflict of Interest

MS and AH have submitted a patent application (DE 102019122014.9) “Inhibition of miR-574-5p as novel therapeutic strategy to reduce bone resorption in arthritis disease.” The remaining authors declare that the research was conducted in the absence of any commercial or financial relationships that could be construed as a potential conflict of interest.
