# Supplementary material for: Extracellular miR-574-5p Induces Osteoclast Differentiation via TLR 7/8 in Rheumatoid Arthritis
Source: Front Immunol. 2020 Oct 14;11:585282. doi: 10.3389/fimmu.2020.585282 (PMC7591713; doi:10.3389/fimmu.2020.585282)
Supplement: Supplementary file 1 [file Data_Sheet_1.PDF]

Supplementary Material

Extracellular miR-574-5p induces osteoclast differentiation via TLR 7/8 in rheumatoid arthritis

Anett B. Hegewald[1], Kai Breitwieser[1], Sarah M. Ottinger[1], Fariborz Mobarrez[2], Marina Korotkova[2], Bence Rethi[2], Per-Johan Jakobsson[2], Anca I. Catrina[2], Heidi Wähämaa[2], Meike J. Saul[1,3]*

[1] Department of Biology, Technische Universität Darmstadt, 64287 Darmstadt, Germany

[2] Rheumatology Unit, Department of Medicine, Solna, Karolinska Institutet, Karolinska University Hospital, SE- 17176 Stockholm, Sweden

[3] Institute of Pharmaceutical Chemistry, Goethe Universität Frankfurt, 60483 Frankfurt am Main, Germany

*** Correspondence:**
Meike J. Saul; Department of Biology, Technische Universität Darmstadt, Darmstadt/Germany; saul@bio.tu-darmstadt.de; +49 6151 22005

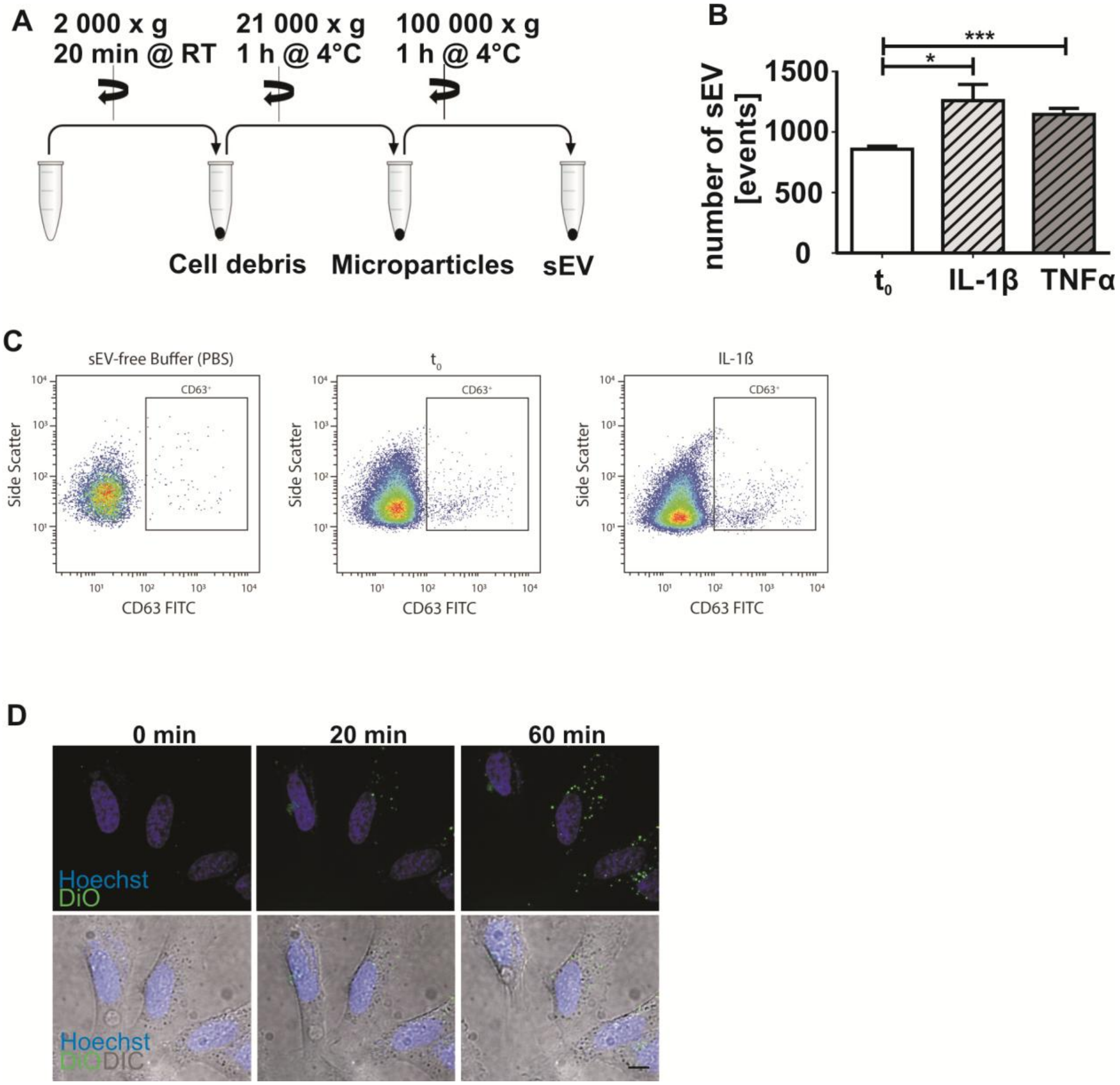

Figure S1: (A) Schematic picture of sEV isolation by different centrifugation steps. (B) FACS analysis of sEV isolated from synovial fibroblast (SFs) cell culture supernatant. SFs from RA patients were cultured in sEV-depleted cell culture medium for 24h. The medium was then replaced by sEV-depleted cell culture medium containing either 10ng/ml IL-1β, or 10ng/ml TNFα for 24h. sEV were isolated by differential ultracentrifugation and analysed by FACS analysis. Data are shown as mean + SEM (N=5), t-test *p <0.05, ***p <0.001. (C) Gating strategy of sEV-FACS analysis. Representative images are shown for PBS control, t_0 and IL-1β samples. (D) miR-574-5p oe sEV uptake of HeLa cells. Purified miR-574-5p oe sEV were labelled with lipophillic tracer 3,3'-dioctadecyloxacarbocy-anine perchlorate (DiO) and applied to HeLa which were stained with 5 µg/ml Hoechst 33258. Images were taken every 10min, scale bar 10µm.

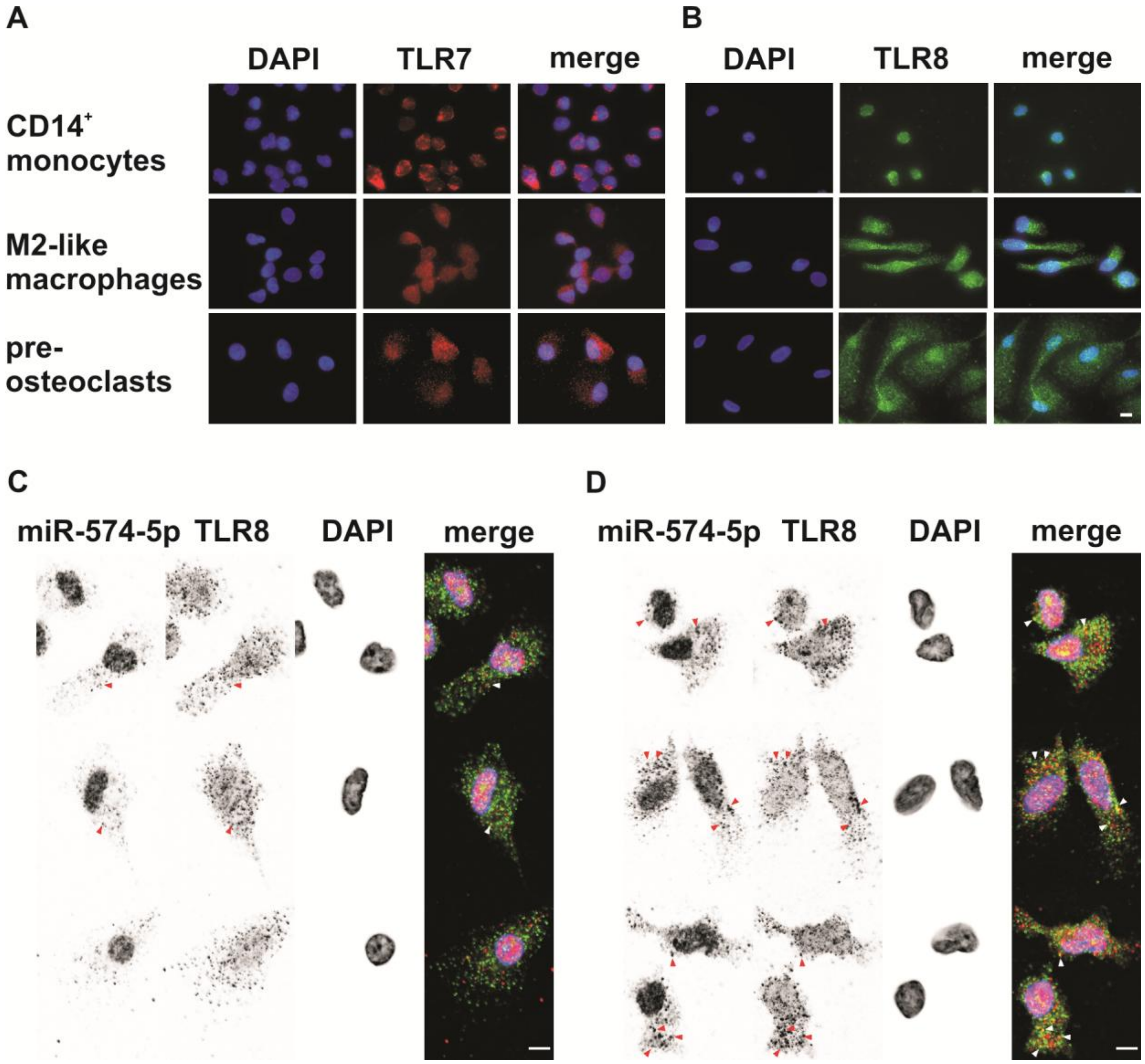

Figure S2: (A/B) TLR7 and TLR8 expression during osteoclastogenesis. Immunofluorescence staining of (A) TLR7 and (B) TLR8 expression. $CD14^+$ monocytes were isolated and differentiated into M2-like macrophages and pre-osteoclasts. Cells were fixed and stained as indicated. Cells were additionally stained with DAPI to visualize the cell nuclei. One representative picture is shown for each staining and cell differentiation status, scale bar = 10µm. (C/D) Combined immunofluorescence and fluorescent *in situ* hybridization of TLR8 and miR.574-5p. $CD14^+$ monocytes were isolated and differentiated into M2-like macrophages for 3 days with M-CSF. (C) Without sEV stimulation or (D) exposed to 4 µg/ml of purified miR-574-5p oe sEV for 15 min. Cells were fixed, epitopes were detected as indicated and nuclei were counterstained with DAPI. Scale bar = 5 µm

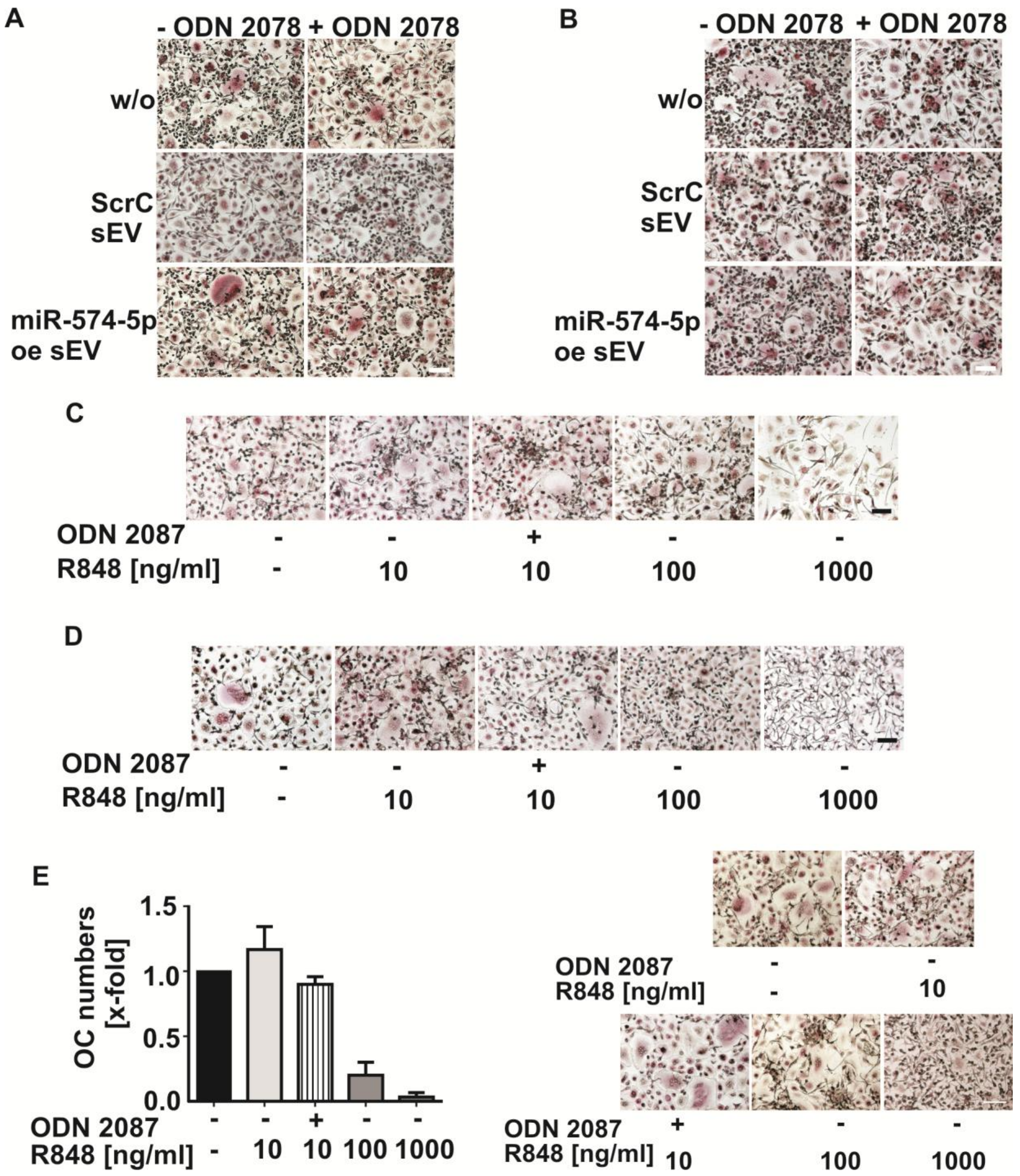

Figure S3: Increased osteoclast (OC) differentiation by sEV delivered miR-574-5p is mediated by TLR7/8 activation. TRAP staining of mature OCs obtained from $CD14^+$ monocytes and cultured in presence of (A/B) 1µg/ml ScrC or miR-574-5p oe sEV and 200 nM ODN 2087 TLR7/8 inhibitor or (C/D) different concentrations of the TLR7/8 ligand R848 (10–1000ng/ml), which were added either to (A/C) $CD14^+$ monocytes or (B/D) M2-like macrophages. Multinucleated cells with three or more nuclei that were stained with a purple colour were considered as OCs. One representative picture is shown for each condition, scale bar 50µm. (E) TRAP staining of mature OCs obtained from $CD14^+$

monocytes and cultured in presence of different concentrations of the TLR7/8 ligand R848 which was given at the stage of pre-osteoclasts. The relative changes normalized to untreated control cells are given as mean + SEM (N=3). Representative images of TRAP positive cells are shown, scale bar 100µm.

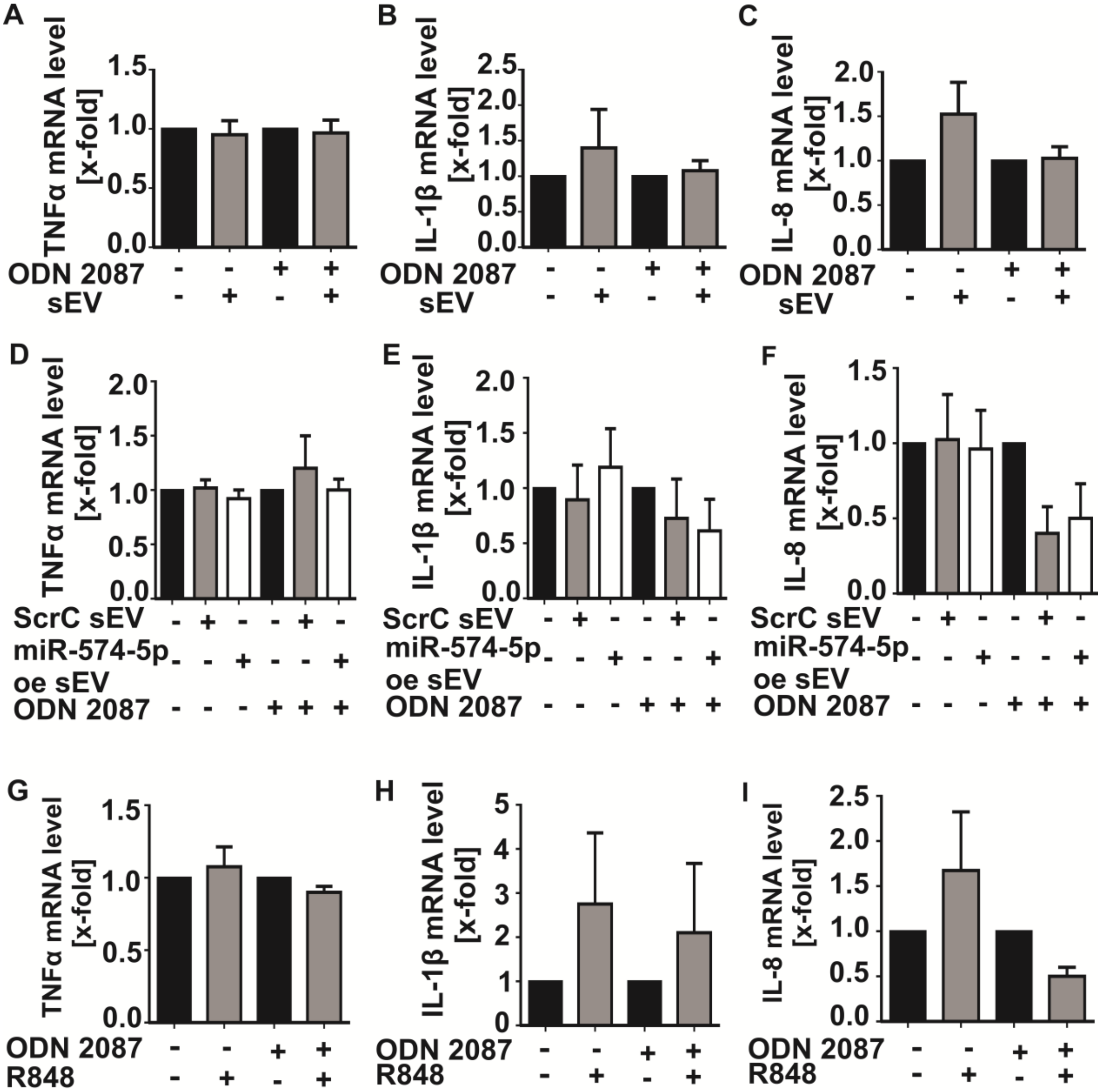

Figure S4: (A-C) Effect of sEV isolated from synovial fluid on TNFα, IL-8, and IL-1β mRNA levels. CD14$^+$ monocytes were stimulated with 4µg/ml sEV isolated from synovial fluid of ACPA$^+$ RA patients and 200nM ODN 2087. Cells were harvested after 4 h of incubation and total RNA was extracted. Quantification of (A) TNFα, (B) IL-1β and (C) IL-8 mRNA levels using RT-qPCR. β-Actin was used as endogenous control. Relative changes normalized to untreated controls are given as + SEM of (N=4). (D-F) Effect of sEV delivered miR-574-5p TNFα, IL-8, and IL-1β mRNA levels. CD14$^+$ monocytes were stimulated with either 1 µg/ml of ScrC or miR-574-5p oe sEV and 200nM ODN 2087. Cells were harvested after 4 h of incubation and total RNA was extracted. Quantification of (D) TNFα, (E) IL-1β and (F) IL-8 mRNA levels using RT-qPCR. (G-I) CD14$^+$ monocytes were stimulated with 10 ng/µl R848 and 200nM of ODN 2087 for 4 h. Total RNA was extracted and RT-qPCR was performed to quantify (G) TNFα, (H) IL-1β and (I) IL-8 mRNA level. β-Actin was used as endogenous control. Relative changes normalized to untreated controls are given as + SEM (N=4).

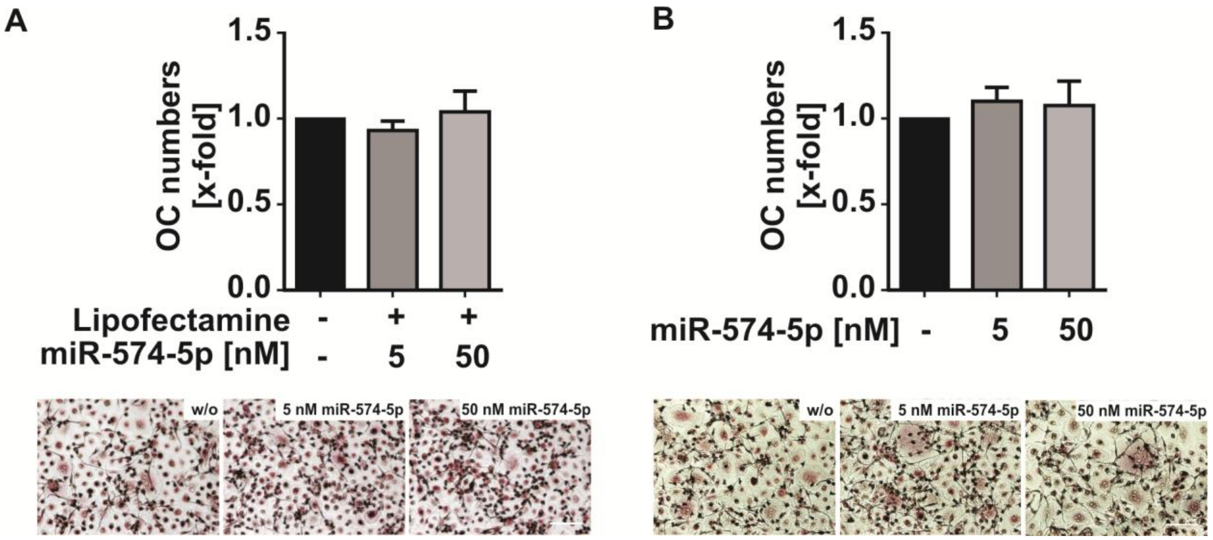

Figure S5: Effect of synthetic miR-574-5p on osteoclast (OC) differentiation Tartrate-resistant acid phosphatase (TRAP) staining of mature OCs obtained from $CD14^+$ monocytes and cultured in presence of (A) 1µg/ml Lipofectamine® 2000 and synthetic miR-574-5p (5 nM or 50nM) or (B) synthetic miR-574-5p alone (5nM or 50nM). To ensure a possible effect, we used an experimental setup with an excess of the naturally occurring content of miR in EVs isolated from synovial fluid. Multinucleated cells with three or more nuclei that were stained with a purple colour were considered as OCs. The relative changes normalized to (A) Lipofectamine® 2000 or (B) untreated control cells are given as mean + SEM (N=3). Representative images of TRAP positive cells are shown below, scale bar 100 µm.
